# Biological pathway‐based Alzheimer’s Disease polygenic risk score and proteinopathy

**DOI:** 10.1002/alz.091359

**Published:** 2025-01-03

**Authors:** Yuriko Katsumata, Xian Wu, Khine Zin Aung, Inori Tsuchiya, Lincoln MP Shade, Shama D Karanth, Kathryn Gauthreaux, Charles Mock, Walter A. Kukull, Erin L. Abner, Peter T Nelson, David W. Fardo

**Affiliations:** ^1^ College of Public Health, University of Kentucky, Lexington, KY USA; ^2^ Sanders‐Brown Center on Aging, University of Kentucky, Lexington, KY USA; ^3^ College of Medicine, University of Florida, Gainesville, FL USA; ^4^ National Alzheimer’s Coordinating Center, University of Washington, Seattle, WA USA; ^5^ College of Medicine, University of Kentucky, Lexington, KY USA

## Abstract

**Background:**

We recently reported genetic associations with dementia‐related proteinopathies. Using multidimensional generalized partial credit modeling, we constructed three continuous latent variables, corresponding to TDP‐43, Aβ/Tau, and a‐synuclein related neuropathology endophenotype scores.

**Method:**

Participant data were drawn from the National Alzheimer’s Coordinating Center (NACC) neuropathology (NP) data (from the September 2023 data freeze) linked to Alzheimer’s Disease Genetics Consortium (ADGC) genotype data. ADGC genotype data were imputed by TOPMed Imputation Server (https://imputation.biodatacatalyst.nhlbi.nih.gov/). The AD summary statistics were obtained from GWAS Catalog (https://ftp.ebi.ac.uk/pub/databases/gwas/summary_statistics/GCST90027001‐GCST90028000/GCST90027158/) created by Bellenguez et al. We calculated biological pathway‐based PRS by Gene Ontology (GO) terms listed for *Homo sapiens* from Ensembl (http://www.ensembl.org/biomart/martview/) with the subset of retained SNPs based on r^2^ = 0.1 with a physical distance threshold of 250 kb and p‐value < 0.0001 using PRSice‐2 (https://www.prsice.info/). We then estimated the associations between each of the PRS scores and each of the three latent proteinopathy scores adjusting for age at death, sex, the top three principal components, and the two non‐outcome latent scores than the outcome. We adjusted the p‐values using the Bonferroni correction.

**Result:**

The total 19,596,813 single nucleotide variants (SNVs) or short indels were overlapped in ADGC imputed genotype and the GWAS summary statistics. We extracted 1,850 biological process, 542 molecular function, and 405 cellular component GO terms that were constructed of 5 or more SNVs. The AD PRSs in “membrane” cellular component and “protein binding” molecular function were strongly associated with the Ab/Tau pathology related latent score (Bonferroni corrected p‐value = 1.7 × 10^‐6^ and 0.00011, respectively). There was no biological pathway‐based AD PRS associated with TDP‐43 and α‐synuclein pathology‐related latent scores.

**Conclusion:**

The biological pathway‐based AD PRSs were associated with only Aβ/Tau pathway related latent proteinopathy score. We will explore PRSs for other diseases that are potentially related to AD etiology including Parkinson’s disease linked to α‐synuclein pathology, amyotrophic lateral sclerosis (ASL) with TDP‐43 aggregation, cardiovascular diseases, cardiovascular risk factors, and psychiatric diseases.

